# Upadacitinib for Immune Checkpoint Inhibitor–Related Dermatitis: A Nonrandomized Clinical Trial

**DOI:** 10.1001/jamaoncol.2026.0136

**Published:** 2026-03-05

**Authors:** Chengshui Chen, Xinyu Liang, Zheng Peng, Yan Zou, Huijuan He, Xiaoyan Zhang, Yi He, Shixiu Wu

**Affiliations:** 1 The Quzhou Affiliated Hospital of Wenzhou Medical University, Quzhou People’s Hospital, Quzhou, China; 2 Zhejiang Engineering Research Center for Intelligent Manufacturing of Clinical Diagnostic Equipment, Hangzhou, China

## Abstract

This nonrandomized clinical trial examines the safety and efficacy of oral upadacitinib in the treatment of patients with severe immune checkpoint inhibitor–related dermatitis.

The conventional primary treatment for severe immune checkpoint inhibitor (ICI)–related dermatitis involves high-dose systemic corticosteroids.^1^ Corticosteroids usually pose significant risks, such as severe infection and gastrointestinal bleeding. Additionally, they attenuate the antitumor efficacy of ICIs.^2^ Upadacitinib, a selective Janus kinase (JAK) 1 inhibitor, suppresses JAK1 in the JAK-STAT pathway. It is indicated for autoimmune and atopic dermatitis.^3^ Herein, the study investigators designed a phase 2 clinical trial to evaluate the resolution rate of patients with various solid tumors who have developed severe (grades 3-4) ICI-related dermatitis.

## Methods

This single-center, open-label, single-group phase 2 nonrandomized clinical trial was conducted at Quzhou People’s Hospital in Zhejiang, China, with approval from the institutional review board and an independent ethics committee (NCT06715982). All participants provided written informed consent. The study was designed as a Simon 2-stage clinical trial. All analyses were conducted using R version 4.2.0 (R Foundation). The protocol and statistical analysis plan are provided in Supplements 1 and 2, respectively.

Patients diagnosed with grades 3 to 4 ICI-related dermatitis received oral upadacitinib (15 mg/d) for 28 days, without taking concomitant glucocorticoids, immunosuppressants, or antihistamines. Patients whose dermatitis was relieved (defined as a reduction to grade ≤1) were permitted to resume their original antitumor therapy.

The primary end points were upadacitinib safety and ICI-related dermatitis resolution. Resolution was defined as grade 1 or less rashes by day 28. Grade 0 indicated complete resolution, while grade 1 demonstrated partial resolution. Adverse events (AEs) were recorded. Secondary end points included the percentage of patients continuing ICIs at day 28 and the change in pruritus severity assessed by the Peak Pruritus Numerical Rating Scale. The 95% CIs were calculated using the Jennison-Turnbull method and a 2-sided α level was set at .05.

## Results

From February to July 2025, 33 of 50 screened patients were enrolled. During the first stage (February-April 2025), the predefined criterion for trial continuation was met, with rash resolution observed in all 14 initial participants. This prompted subsequent enrollment of 19 additional patients in the second stage (April-August 2025). All patients were treatment-naive, with no prior exposure to corticosteroids and other immunosuppressants (Table 1).

**Table 1.** Characteristics of Patients With Immune Checkpoint Inhibitor (ICI)–Related Dermatitis at Diagnosis

Characteristic	No. of patients (%) (N = 33)
Age, median (range), y	61 (25-86)
Sex	
Male	19 (57.6)
Female	14 (42.4)
Cancer type	
Lung	11 (33.3)
Gallbladder carcinoma	3 (9.1)
Gastric	4 (12.1)
Colorectal	3 (9.1)
Cervical	3 (9.1)
Hypopharyngeal	1 (3.0)
Cholangiocarcinoma	2 (6.1)
Liver	1 (3.0)
Esophageal	5 (15.2)
ICI	
PD-1 monoclonal antibody	13 (39.4)
PD-L1 monoclonal antibody	11 (33.3)
CTLA-4/PD-1 bispecific antibody	9 (27.3)
Dermatitis grade	
3	29 (87.9)
4	4 (12.1)
ICI cycles	
≤4	10 (30.3)
5-11	15 (45.5)
≥12	8 (24.2)
Rash presentation	
Maculopapular	26 (78.8)
Lichenoid	2 (6.1)
Psoriasiform	4 (12.1)
Toxic epidermal necrolysis	1 (3.0)
Pruritus	
Positive	33 (100.0)
Negative	0
PP-RNS score	
1-3	0
4-6	10 (30.3)
7-8	13 (39.4)
9-10	10 (30.3)
Rash relief	
Complete resolution (grade 0)	30 (90.9)
Partial resolution (grade 1)	3 (9.1)

Abbreviations: CTLA-4, cytotoxic T-lymphocyte antigen 4; PD-1, programmed cell death 1; PD-L1, programmed cell death ligand 1; PP-RNS, Peak Pruritus Numerical Rating Scale.

Overall, 9 patients (27.3%) experienced 1 or more upadacitinib-related AE. The most common AE was creatine kinase elevation, observed in 5 patients (15.2%). No serious AEs (grades 3-5) related to upadacitinib were observed. No patients discontinued upadacitinib due to AEs. Table 2 summarizes upadacitinib-related AEs.

**Table 2.** Overview of Adverse Events (AEs) During the Treatment and Follow-Up Periods

AE	No. (%)					
	Related to antitumor or other therapy (n = 33)			Related to upadacitinib (n = 33)		
	Grades 1-2	Grades 3-4	Grade 5	Grades 1-2	Grades 3-4	Grade 5
Any AE	102 (309.1)^a^	17 (51.5)	0	12 (36.4)	0	0
Any patients with AEs	33 (100.0)	13 (39.4)	0	9 (27.3)	0	0
AEs of special interest						
Hepatic disorder	7 (21.2)	1 (3.3)	0	0	0	0
Anemia	6 (18.2)	2 (6.7)	0	0	0	0
Cough	6 (18.2)	0	0	0	0	0
Constipation	9 (27.3)	1 (3.3)	0	0	0	0
Diarrhea	4 (12.1)	2 (6.7)	0	0	0	0
Fatigue	13 (39.4)	2 (6.7)	0	4 (12.1)	0	0
Fever	3 (9.1)	0	0	0	0	0
Leukopenia	10 (30.3)	3 (9.1)	0	0	0	0
Lymphopenia	17 (51.5)	2 (6.7)	0	0	0	0
Nausea or vomiting	9 (27.3)	0	0	0	0	0
Neutropenia	8 (24.2)	4 (12.1)	0	0	0	0
Acne	0	0	0	3 (9.1)	0	0
Creatine kinase elevation	4 (12.1)	0	0	5 (15.2)	0	0
Kidney function disorder	3 (9.1)	0	0	0	0	0
Tuberculosis	0	0	0	0	0	0
Infections	3 (9.1)	0	0	0	0	0
Thrombotic events	0	0	0	0	0	0
Cardiovascular events	0	0	0	0	0	0
Gastrointestinal perforation	0	0	0	0	0	0

^a^ Patients could experience more than 1 AE.

After initiation of upadacitinib, all patients reported significant rash relief. The resolution rate was 100% (95% CI, 89%-100%) at day 28. Rash resolution occurred within 3 to 5 days in 51.5% of patients (17 of 33), 5 to 7 days in 18.2% (6 of 33), and 7 to 14 days in 9.1% (3 of 33). Given the rapid relief of skin rashes, 31 patients (93.9%) continued ICI treatment as scheduled, while 2 patients with ICI-related dermatitis (grade 4) did not. All patients reported significant improvement in pruritus within 1 day, with a reduction of more than 4 points on the Peak Pruritus Numerical Rating Scale.

## Discussion

To the study investigators’ knowledge, this is the first clinical trial to investigate the use of oral JAK inhibitors for ICI-related dermatitis. Most patients in the study responded quickly to upadacitinib, with pruritus almost disappearing within 1 day after treatment initiation, skin rashes significantly relieved within 4 days, and near-complete resolution (grade ≤1) within 7 days. In contrast, the median time to resolution for ICI-related dermatitis treated with systemic corticosteroids and other immunosuppressants was reported to be 55 days.^4^

AEs were significantly fewer than those in previous upadacitinib studies, with no serious AEs observed.^5^ This may be attributable to the low dose and short regimen in this study.

Limitations included the single-group design, which precluded randomized control; small sample size; and lack of long-term follow-up.

In conclusion, upadacitinib could be a potential therapeutic option for managing ICI-related dermatitis. Nevertheless, future randomized clinical trials are warranted.

## Notes

Supplement 1. Trial Protocol

Supplement 2. Statistical Analysis Plan

Supplement 3. Data Sharing Statement
