# Supplementary material for: Upadacitinib for Immune Checkpoint Inhibitor–Related Dermatitis: A Nonrandomized Clinical Trial
Source: JAMA Oncol. 2026 Mar 5;12(5):526–8. doi: 10.1001/jamaoncol.2026.0136 (PMC12964249; doi:10.1001/jamaoncol.2026.0136)
Supplement: Supplement 1. — Trial Protocol [file jamaoncol-e260136-s001.pdf]

Protocol

Protocol for:

This trial protocol has been provided by the authors to give readers additional information about the work.

This supplement contains the following items:

1. Original protocol (V1.0).

JAK inhibitors for Immune Checkpoint Inhibitors-Related Dermatitis: An Open-Label, Single-Arm, Phase II Clinical Trial

Protocol Number:	HangCH051
Version No.:	1.0
Version Date:	2024-11-28
Investigator:	Shixiu Wu
Study Site:	Quzhou people's hospital, Zhejiang province, China
Contact:	Shixiu Wu
Contact Information:	05703123031

Index

Synopsis 4
1 Study Title 7
2 Study Background and Rationale 7
2.1 Introduction of ICI-related dermatitis 7
2.2 Study Rationale 7
3 Study Objectives 8
3.1 Primary Objective 8
3.2 Secondary objective 8
3.3 Primary study endpoints 8
3.4 Secondary study endpoints 9
4 Expected Effect 9
5 Study Design 9
6 Subject Inclusion, Exclusion Criteria, and Assignment Method of Enrollment 9
6.1 Subject Inclusion Criteria 9
6.2 Exclusion criteria 10
7 Number of Subjects Enrolled 10
8 Test Items for Subjects before and after Treatment 10
9 Discontinuation from Study Drug or Subject Withdrawal from Study 11
10 Study Drug 11
11 Management of Dropouts 12
12 Conditions for Study Interruption 12
13 Conditions for Study Discontinuation 13
14 Requirements for Recording Adverse Events and Procedures for Reporting and Managing Serious Adverse Events 14
14.1 Recording Requirements for Adverse Events 14
14.2 Reporting Methods and Management Measures of Serious Adverse Events 14
15 Statistical Analysis of Study Results 15
15.1 Statistical Analysis Principles 15
15.2 Sample Size Calculation 15
15.3 Statistical Analysis Methods 15
15.4 Statistical Analysis Population 16
16 Follow-up Plan and Implementation Measures 16
16.1 Follow-up Plan 16
16.2 Implementation Method 17
17 Ethical Requirements 18
18 Quality Control and Quality Assurance 18
18.1 Quality Control 18
19 Publishing of Articles 18

Synopsis

**Protocol Number**	HangCH051
**Study Title**	JAK inhibitors may be the optimal option for ICI-related dermatitis ---A open Label, phase II clinical trial
**Sponsor**	Quzhou people’s hospital, Zhejiang province, China
**Testing Facility**	Quzhou people’s hospital, Zhejiang province, China
**Study Phase**	Phase II
**Indication**	Adult patients with ICI-related dermatitis
**Study Objectives**	**Primary Study Objectives:**<br>● Evaluate the safety of JAK inhibitors (JAKi) in adult patients with ICI-related dermatitis.<br>● Explore the efficacy of JAKi in adult patients with ICI-related dermatitis.<br><br>**Other Study Objective:**<br>● Explore the proportion of continued ICIs utilization at 28 days<br>● Explore the change of pruritus severity
**Study Endpoints**	**Primary study endpoints:**<br>The primary endpoints were the safety and efficacy of JAK inhibitors for ICI-related dermatitis.<br>● Evaluate the safety of JAK inhibitors in adult patients with ICI-related dermatitis. The safety will be assessed based on the incidence and severity of adverse events (AEs) and serious adverse events (SAEs) during upadacitinib treatment. The severity of AEs will be graded using NCI CTCAE v5.0.<br>● The efficacy evaluated by the proportion of patients achieving relief from rashes (defined as ICI-related dermatitis grade ≤1 according to CTCAE v5.0.<br><br>**Secondary study endpoints:**<br>● Explore the proportion of continued ICIs utilization at 28 days

	● Explore the change of pruritus severity assessed by Peak Pruritus Numerical Rating Scale (PP-NRS), score 0-10, a higher score indicates a more severe pruritus condition
**Study Design**	**Overall study design:**<br>● This is a Simon two-stage clinical trial. The historical response rate of corticosteroids is 65%, the expected response rate is 85% , with α= 0.05 and β=0.8. A sample size of 14 cases is planned for enrollment in the first phase. The study will proceed to the next phase if the number of patients with effective treatment is > 10; otherwise, it will be terminated. The second phase is designed to enroll 19 patients. If the total number of effective responses exceeds 25, indicating that the treatment is effective. A 5%-10% dropout rate was considered in the sample size calculation, resulting in a total sample size of 35 subjects. The trial included a 4-week treatment and a 1-month follow-up period. All enrolled patients received upadacitinib 15mg exclusively for 28 days. During this period, no concomitant use of glucocorticoids, immunosuppressants, or antihistamines was permitted. If ICI-related dermatitis achieved relief (defined as ICI-related dermatitis grade $\leqslant$1 according to CTCAE v5.0), antitumor therapy could be resumed according to the patient's original treatment plan. α = 0.05 and β =0.8
**Total number of subjects**	Approximately 35 subjects
Inclusion Criteria	1. Eligible patients must be at least 18 years of age with a confirmed diagnosis of a solid malignant tumor.<br>2. Patients who have received treatment with any Food and Drug Administration (FDA)-approved monoclonal antibodies targeting CTLA-4, PD-1, or PD-L1, either as monotherapy or in combination.<br>3.Patients who are diagnosed with Immune checkpoint inhibitors (ICI)-related dermatitis graded as 3-4 according to Common Terminology Criteria for Adverse Events Version 5.0.<br>4. Patients with ICI-related dermatitis who were either treatment-naïve (having received no prior steroids or immunosuppressants) or were

	refractory to previous treatment with corticosteroids and/or immunosuppressive agents.<br>5. Adequate bone marrow and organ function, as outlined below, must be confirmed:<br>1) White blood cell (WBC) count >= 2.0 × 10^9/L 2) Absolute neutrophil count (ANC) >= 1.5 × 10^9/L 3) Platelet count (PLT) >= 75 × 10^9/L 4) Hemoglobin (Hgb) >= 90 g/L 5) AST and ALT <= 3 × upper limit of normal (ULN) in patients without hepatic metastases; <= 5 × ULN in those with hepatic metastases, provided the elevation is not attributable to ICI-related hepatitis 6) Total bilirubin <= 2 × ULN, except in cases of Gilbert's syndrome (where total bilirubin must be < 3.0 mg/dL), and not due to ICI-related hepatotoxicity<br>6. All participants must be capable of providing personally signed and dated informed consent, demonstrating understanding of all relevant study aspects.
**Exclusive criteria**	1.Patients with known underlying medical conditions (e.g., chronic inflammatory skin disorders such as atopic dermatitis or psoriasis) that, in the investigator's assessment, may elevate the risks associated with study participation or compromise the interpretation of study outcomes.<br>2.Patients who currently present with persistent dermatitis (grade >1, according to CTCAE v5.0) attributed to therapeutic interventions other than ICIs treatment.<br>3. Patients in pregnancy.<br>4. Confirmed infection with human immunodeficiency virus (HIV), hepatitis B virus (HBV), hepatitis C virus (HCV).<br>5.Any other medical, psychiatric, or logistical condition that, in the judgment of the investigator, could pose a safety risk, affect protocol compliance, or interfere with the conduct or interpretability of the study.
**Study Drug**	Upadacitinib (15mg once daily for 28days)
**Safety Evaluation**	The safety assessment measures include: physical examination, vital signs, electrocardiograph (ECG), laboratory tests (such as hematology, blood chemistry, and urinalysis), and adverse events.
**Sample Size Estimation**	● This is a Simon two-stage clinical trial. The historical response rate of corticosteroids is 65%, the expected response rate is 85% , with α=

	0.05 and β=0.8. The study will proceed to the next phase if the number of patients with effective treatment is > 10; otherwise, it will be terminated. The second phase is designed to enroll 19 patients. If the total number of effective responses exceeds 25, indicating that the treatment is effective. A 5%-10% dropout rate was considered in the sample size calculation, resulting in a total sample size of 35 subjects.

1 Study Title

JAK inhibitors for Immune Checkpoint Inhibitors-Related Dermatitis: An Open-Label, Single-Arm, Phase II Clinical Trial

2 Study Background and Rationale

2.1 Introduction of ICI-related dermatitis

The widespread clinical use of immune checkpoint inhibitors (ICIs) has led to a substantial improvement in overall survival rates among patients diagnosed with various solid tumors, including malignancies of the lung, breast, esophagus, liver, stomach, colorectum, as well as melanoma.[1] Nevertheless, ICI-related adverse events (irAEs) emerge in nearly 70% of recipients, primarily as a consequence of systemic immune activation. Among these side effects, dermatitis is one of the most frequently reported toxicities, affecting between 30% and 60% of individuals undergoing therapy, and can necessitate the interruption of immunotherapy. In its most severe forms, this cutaneous reaction may become life-threatening[2-5].

2.2 Study Rationale

As outlined in the NCCN guidelines, systemic corticosteroids are recommended as first-line agents for the management of dermatitis associated with ICIs[4]. Nonetheless, treatment of this condition remains clinically challenging due to the limited response observed in certain patient cohorts following conventional corticosteroid regimens. Moreover, corticosteroid therapy is associated with substantial risks, including increased vulnerability to infections, gastrointestinal adverse events, metabolic dysregulation, and neuropsychiatric symptoms[6]. Of particular concern is the potential for corticosteroids to diminish the antitumor activity of ICIs, creating a paradoxical clinical dilemma. Accumulating evidence suggests that corticosteroid use may drive tumor progression, enhance metastatic potential, and correlate with reduced overall survival in oncologic patients. These concerns emphasize the necessity of developing novel

treatment modalities that effectively manage dermatitis without compromising antitumor immunity and with improved safety profiles. The optimal intervention would maintain the efficacy of ICIs while avoiding considerable treatment-induced toxicity[7].

The molecular mechanisms underlying ICI-induced dermatitis have not been fully elucidated, and few dedicated studies are available in this area. Based on our experimental data and previous reports implicating the JAK/STAT signaling pathway in autoimmune cutaneous disorders—such as atopic dermatitis, vitiligo, psoriasis—and severe cutaneous adverse reactions like toxic epidermal necrolysis (TEN[8]), we propose that this pathway may also contribute to the development of ICI-related dermatitis. JAK inhibitors, which target key upstream events in JAK/STAT activation, function through pan-cytokine inhibition leading to immunosuppression[9], and are already validated for use in several immune-mediated dermatologic diseases. Although several cases have reported promising outcomes with JAK inhibitors for steroid-refractory irAEs, well-controlled clinical trials focusing specifically on dermatitis are still lacking[10].

3 Study Objectives

3.1 Primary Objective

1) Evaluate the safety of JAK inhibitors (JAKi) in adult patients with ICI-related dermatitis.
2) Explore the efficacy of JAKi in adult patients with ICI-related dermatitis.

3.2 Secondary objective

1)Explore the proportion of continued ICIs utilization at 28 days
2)Explore the change of pruritus severity

3.3 Primary study endpoints

The primary endpoints were the safety and efficacy of JAK inhibitors for ICI-related dermatitis.

1) Evaluate the safety of JAK inhibitors in adult patients with ICI-related dermatitis. The safety will be assessed based on the incidence and severity of adverse events (AEs) and serious adverse events (SAEs) during upadacitinib treatment. The severity of AEs will be graded using NCI CTCAE v5.0.

2) The efficacy evaluated by the proportion of patients achieving relief from rashes (defined as ICI-related dermatitis grade ≤1 according to CTCAE v5.0,).

3.4 Secondary study endpoints

1)Explore the proportion of continued ICIs utilization at 28 days
2)Explore the change of pruritus severity assessed by Peak Pruritus Numerical Rating Scale (PP-NRS), score 0-10, a higher score indicates a more severe pruritus condition

4 Expected Effect

The expected response rate is 85%

5 Study Design

This single-center, open-label, single-arm phase II clinical trial was conducted at Quzhou People's Hospital in Zhejiang Province, China. This is a Simon two-stage clinical trial. The historical response rate of corticosteroids is 65%, the expected response rate is 85% , with α= 0.05 and β=0.8. A sample size of 14 cases is planned for enrollment in the first phase. The study will proceed to the next phase if the number of patients with effective treatment is > 10; otherwise, it will be terminated. The second phase is designed to enroll 19 patients. If the total number of effective responses exceeds 25, indicating that the treatment is effective. A 5%-10% dropout rate was considered in the sample size calculation, resulting in a total sample size of 35 subjects. The trial included a 4-week treatment and a 1-month follow-up period. All enrolled patients received upadacitinib 15mg exclusively for 28 days. During this period, no concomitant use of glucocorticoids, immunosuppressants, or antihistamines was permitted. If ICI-related dermatitis achieved relief (defined as ICI-related dermatitis grade ≤1 according to CTCAE v5.0), antitumor therapy could be resumed according to the patient's original treatment plan.

6 Subject Inclusion, Exclusion Criteria, and Assignment Method of Enrollment

6.1 Subject Inclusion Criteria

1. Eligible patients must be at least 18 years of age with a confirmed diagnosis of a solid malignant tumor.

2. Patients who have received treatment with any Food and Drug Administration (FDA)-approved monoclonal antibodies targeting CTLA-4, PD-1, or PD-L1, either as monotherapy or in combination.

3.Patients who are diagnosed with Immune checkpoint inhibitors (ICI)-related dermatitis

graded as 3-4 according to Common Terminology Criteria for Adverse Events Version 5.0.

4. Patients with ICI-related dermatitis who were either treatment-naïve (having received no prior steroids or immunosuppressants) or were refractory to previous treatment with corticosteroids and/or immunosuppressive agents.

5. Adequate bone marrow and organ function, as outlined below, must be confirmed:

1) White blood cell (WBC) count >= 2.0 × 10^9/L 2) Absolute neutrophil count (ANC) >= 1.5 × 10^9/L 3) Platelet count (PLT) >= 75 × 10^9/L 4) Hemoglobin (Hgb) >= 90 g/L 5) AST and ALT <= 3 × upper limit of normal (ULN) in patients without hepatic metastases; <= 5 × ULN in those with hepatic metastases, provided the elevation is not attributable to ICI-related hepatitis 6) Total bilirubin <= 2 × ULN, except in cases of Gilbert's syndrome (where total bilirubin must be < 3.0 mg/dL), and not due to ICI-related hepatotoxicity

6. All participants must be capable of providing personally signed and dated informed consent, demonstrating understanding of all relevant study aspects.

6.2 Exclusion criteria

1.Patients with known underlying medical conditions (e.g., chronic inflammatory skin disorders such as atopic dermatitis or psoriasis) that, in the investigator's assessment, may elevate the risks associated with study participation or compromise the interpretation of study outcomes.

2.Patients who currently present with persistent dermatitis (grade >1, according to CTCAE v5.0) attributed to therapeutic interventions other than ICIs treatment.

3. Patients in pregnancy.

4. Confirmed infection with human immunodeficiency virus (HIV), hepatitis B virus (HBV), hepatitis C virus (HCV).

5.Any other medical, psychiatric, or logistical condition that, in the judgment of the investigator, could pose a safety risk, affect protocol compliance, or interfere with the conduct or interpretability of the study.

7 Number of Subjects Enrolled

Approximately 35 subjects.

8 Test Items for Subjects before and after Treatment

1) Basic examination: height, weight, blood pressure, heart rate, etc.

2) Hematology: hematology, blood chemistry, etc.

3) Virological test: Includes testing for hepatitis viruses (such as HAV, HBV, HCV), HIV, Epstein-Barr virus, syphilis-causing spirochete (Treponema pallidum), and Helicobacter pylori, etc.

4) Immune-related tests: antinuclear antibody and ENA antibody spectrum tests to rule out Autoimmune Disease, will be conducted before screening.

5)12-lead ECG, 24-h Holter if necessary.

6) Chest x-ray, and abdominal ultrasound examination (including the liver, spleen and other organs).

9 Discontinuation from Study Drug or Subject Withdrawal from Study

Subjects can request to be discontinued from participating in the study at any time for any reason including but not limited to disease progression or lack of response to treatment. The investigator may discontinue any subject's participation at any time for any reason, including but not limited to disease progression, lack of response to treatment, an AE, safety concerns, or failure to comply with the protocol. Subjects will have study drug discontinued immediately if any of the following occur:

1) Clinically significant abnormal laboratory results or AEs that rule out continuation of the study drug, as determined by the investigator.

2) Serious infections (e.g., sepsis) that cannot be adequately controlled by anti-infective treatment or would put the subject at risk for continued participation in the trial as determined by the investigator.

3) The investigator believes it is in the best interest of the subject.

4) Subject develops a gastrointestinal perforation.

5) Inclusion or exclusion criteria violation was noted after the subject started the study drug, when continuation of the study drug would place the subject at risk as determined by the investigators.

6) Introduction of prohibited medications or dosages when continuation of the study drug would place the subject at risk, as determined by the investigators.

7) The subject becomes pregnant while on study drug.

8) Subject is significantly non-compliant with study procedures, which would put the subject at risk for continued participation as determined by the investigators.

10 Study Drug

Study drug will be taken orally once daily beginning on Day 1 (Baseline) and should be

taken at approximately the same time each day, with or without food. Subjects will be instructed to return all drug containers (even if empty) to the study site personnel at each study visit; study site personnel will document compliance. Investigators will supply upadacitinib. All study drug (IP and CS) must be stored at controlled room temperature (15° to 25°C/59° to 77°F).

Study drug will be packaged in quantities sufficient to accommodate study design. In the event of AEs during the study, meticulous observation is essential, and the investigator should provide the necessary supportive treatment based on their clinical experience and/or

11 Management of Dropouts

Regardless of the reason for withdrawal, case record forms should be retained for all subjects who exit the study. The last test result should be used as the final result, which will then be used for safety or efficacy analysis.

12 Conditions for Study Interruption

The term "study interruption" refers to the cessation of all clinical studies before they have been completed as per the original plan. The primary objective of interrupting a study is to safeguard the rights and interests of the subjects, ensure the quality of the study, and prevent unnecessary financial losses.

1) In case of serious safety concerns arising during the study, it is imperative to promptly interrupt the study.

2) If the drug therapy proves to be excessively ineffective or entirely non-beneficial, demonstrating no clinical value, interrupting the study is essential. This measure not only prevents the delay of efficacious treatment for the subjects but also mitigates unnecessary financial losses.

3) If major errors are identified in the clinical study protocol, making it challenging to evaluate the drug's effect; or if significant deviations occur during implementation in the case of a well- structured protocol, further evaluation of the drug's effect becomes problematic.

4) If the investigator requests interruption (due to financial reasons, administrative reasons, etc.).

5) Cancellation of the study by the administrative department.

For interruption under these conditions, a final evaluation of the subject should be conducted, and any premature discontinuation of the study should be duly recorded on the relevant page of the Case Report Form (CRF). For subjects who have withdrawn from the study due to AEs, it is incumbent upon the investigator to continue the follow-up.

13 Conditions for Study Discontinuation

In any of the following situations, the National Medical Products Administration has the authority to instruct the applicant to revise the study protocol, or to suspend or discontinue the clinical study:

1) Failure of the Ethics Committee to perform its duties;

2) Failure to effectively ensure the safety of subjects;

3) Failing to report a SAE within the specified time limit;

4) Evidence proving that the clinical study drug is ineffective;

5) The occurrence of quality problems with the clinical study drugs;

6) Arranging and collecting fraudulent clinical study data;

7) Other violations of Good Clinical Practice.

14 Requirements for Recording Adverse Events and Procedures for Reporting and Managing Serious Adverse Events

14.1 Recording Requirements for Adverse Events

In the event of an adverse event following treatment with upadacitinib, incidents involving death or serious injury must be reported to the appropriate monitoring agency. A serious injury is defined as one of the following: 1) a life-threatening situation; 2) permanent impairment of a body function or permanent damage to a body structure; 3) the necessity of medical intervention to prevent such permanent impairment or damage. The investigator is responsible for documenting the subject's symptoms, physical signs, and laboratory test results, as well as the onset, duration, severity, treatment measures, and progression of any injuries, within the original medical records and Case Report Form (CRF). It is imperative that these records are truthful, precise, comprehensive, promptly updated, and compliant with legal requirements. The investigator should also complete the SAE report form, providing a signature and date. Furthermore, the original records should include the time of reporting, the method of reporting (whether by telephone, fax, or in writing), and the institution from which the report is issued.

14.2 Reporting Methods and Management Measures of Serious Adverse Events

1) While monitoring the efficacy, it is crucial to vigilantly observe any AEs or unforeseen toxic side effects (including symptoms, physical signs, and laboratory test results). Analyze the underlying causes, make informed judgments, and ensure diligent tracking, observation, and documentation.

2) During the course of the study, any AEs experienced should be meticulously documented in the Case Report Form (CRF). This documentation should include the symptoms, severity, onset time, duration, and progression of the event. The correlation of these events to the study drug should be thoroughly evaluated. The investigator is responsible for providing a detailed record of these events, which should be signed and dated for verification.

3) Upon the detection of an AE, the attending physician has the discretion to determine whether to cease observation based on the patient's condition. Cases where medication has been discontinued due to an adverse event should be subject to follow-up investigations, with the course of action taken and the outcomes meticulously documented.

4) In the event of a SAE occurring during the clinical study, the institution responsible for the study must promptly implement measures to safeguard the safety of the subjects. Furthermore, they are required to report the incident to the Drug Supervision and Management Bureau, the study sponsor, and the Ethics Committee (EC) within a 24-hour timeframe. The

investigator is obliged to sign and date the report. The sponsor, on their part, will ensure full compliance with all legal and regulatory requirements pertaining to the reporting process.

5) Investigators are required to monitor, observe, and document the progression of all AEs, tracking subjects who have withdrawn from the study due to these events until their complete resolution. It is incumbent upon the investigator to ascertain whether the AE is associated with the study drug, and to provide substantiating evidence for this determination.

6) All clinically significant abnormalities identified in clinical examinations or laboratory tests should be duly recorded in the Adverse Event Form. These cases should be monitored and followed up on a weekly basis at the very least, until they return to normal or reach baseline levels.

15 Statistical Analysis of Study Results

15.1 Statistical Analysis Principles

1) The design of the clinical study, along with the presentation and analysis of its results, is carried out using universally accepted statistical methods throughout all stages of the study.

2) A person familiar with biostatistics is responsible for each step.

3) Any changes in statistical methods will be documented and justified in detail.

4) The presentation of statistical analysis results emphasizes the comprehension of clinical implications. The assessment of efficacy should consider both the variance in confidence intervals and the outcomes of significance testing, rather than solely depending on the latter.

5) A comprehensive description of any data that is missing, unused, or excessive in the statistical analysis will be provided. The statistical report of the clinical study must align with the summary report of the same clinical study.

15.2 Sample Size Calculation

This is a Simon two-stage clinical trial. This is a Simon two-stage clinical trial. The historical response rate of corticosteroids is 65%, the expected response rate is 85% , with α= 0.05 and β=0.8. A sample size of 14 cases is planned for enrollment in the first phase. The study will proceed to the next phase if the number of patients with effective treatment is > 10; otherwise, it will be terminated. The second phase is designed to enroll 19 patients. If the total number of effective responses exceeds 25, indicating that the treatment is effective. A 5%-10% dropout rate was considered in the sample size calculation, resulting in a total sample size of 35 subjects. The trial included a 4-week treatment and a 1-month follow-up period. All enrolled patients received upadacitinib 15mg exclusively for 28 days.

15.3 Statistical Analysis Methods

The patient data will be presented as follows: Quantitative data will be described using the mean ± standard deviation or median (interquartile range); categorical data will be represented by rates or percentages; AEs and safety measures experienced by patients will be detailed separately according to their respective grades. The temporal changes in quantitative indicators will be illustrated through trend lines. Statistical analyses will be conducted using SAS 9.4, SPSS 26.0 or R, version 4.2.0software. Unless otherwise specified, a P-value of ≤ 0.05 is deemed statistically significant.

15.4 Statistical Analysis Population

Intention-to-Treat (ITT): Includes all enrolled subjects.

Full Analysis Set (FAS): Includes all enrolled subjects, who receive at least one dose of the study drug and have at least one valid platelet count after treatment.

Per Protocol Set (PPS): Subset of FAS， includes all subjects who complete the administration according to the protocol with no major protocol violation.

Pharmacodynamics Analysis Set (PDS）: Includes all subjects who receive at least one dose of the study drug and have at least one valid pharmacodynamic data after treatment. Immunogenicity Set (IMGS): Included all patients who receive at least one dose of the study drug and have at least one valid immunogenicity data after treatment.

Safety Set (SS): Includes all subjects who receive at least one dose of the study drug.

All above analysis sets will be discussed and decided jointly by the principal investigator, the sponsor, the statistician, and data management personnel at the data review meeting prior to the database lock.

16 Follow-up Plan and Implementation Measures

16.1 Follow-up Plan

1) Cycle

Weekly for 4 weeks; follow-up will be performed weekly after treatment.

2) Follow-up Contents

Follow-up aims to assess the therapeutic effect, monitor the progression and recovery of the subject's condition post-discharge, guide the subject on when to revisit the hospital for follow- up consultations, provide the course of action following any changes in the subject's condition, and other professional technical advices.

3) Follow-up Period and Record of Results

The follow-up cycle extends for 1-month post-dose, with weekly monitoring of physical examination, hematology tests, liver and kidney function, Vital signs, 12-lead

electrocardiograms, serum chemistry/hematology, high-sensitivity C-reactive protein levels, stool examination and urinalysis. Comprehensive records are maintained, documenting the time and any changes in the patient's test results.

4) Database Update

Upon the conclusion of each follow-up visit, it is imperative to promptly integrate the newly acquired information into the patient's initial diagnosis and treatment record. Concurrently, the associated entries and content of the individual case records within the database should be updated accordingly.

5) Lost to Follow-up

Subjects may be considered lost to follow-up if they cannot be re-contacted due to unique circumstances such as refusal of visit, relocation of household registration, disconnection due to relocation, or being untraceable. If failing to be reached for three consecutive follow-ups, the subject will be deemed as lost to follow-up.

6) Follow-up Discontinuation

Follow-up procedures may be discontinued for subjects who are lost to follow up, as well as for those whose death has been confirmed through the follow-up process.

16.2 Implementation Method

1) Individualized follow-up will be performed.

2) The decision to conduct follow-ups on subjects will be made by the supervising physician, who will meticulously document the timing, content, and results of the follow-up, as well as the decision on whether to continue with further follow-ups. Special circumstances necessitate immediate and ongoing follow-up.

3) Follow-up procedures must be conducted in a standardized and adaptable manner, promptly responding to the subject's medical condition. It is imperative that the follow-up staff should be fully apprised of the most recent medical advices and precise examination results prior to initiating the follow-up process. Any form of subjective conjecture, speculation, or negligence is strictly forbidden.

4) Follow-up procedures must uphold patient confidentiality, demonstrate politeness, utilize appropriate language, maintain a compassionate approach, and adhere to pertinent medical laws.

5) The attending physician for follow-up care should not solely depend on the follow-up information to make medical decisions. Instead, a comprehensive approach should be adopted, taking into account the opinions and assessments of the investigators.

6) Upon learning of the subject's death, it is crucial to exercise discretion in the tone of language used.

17 Ethical Requirements

This clinical study will be carried out in strict adherence to the Declaration of Helsinki (2008 Edition) and the pertinent regulations and standards of Good Clinical Practice within our country. Prior to the commencement of the study, the protocol must receive approval from the Ethics Committee (EC) of the study site.

Prior to the inclusion of each subject in this clinical study, it is incumbent upon the study physician to provide a thorough and comprehensive written explanation of the study's objectives, procedures, and potential risks to the subject or their designated representative. Subjects should be made aware of their right to withdraw from the study at any given time. An Informed Consent Form (ICF) must be provided to each subject before their participation is confirmed. The study physician is responsible for ensuring that each subject signs the ICF prior to their involvement in the clinical study, and that this document will be retained within the study archives.

18 Quality Control and Quality Assurance

18.1 Quality Control

1) Protocol establishment: The clinical study protocol is collaboratively discussed and negotiated by all investigators involved in this study. Following a consensus on revisions, the application should be submitted to the Ethics Committee (EC) for review and approval.

2) The criteria for determining abnormalities in laboratory tests are established according to the normal reference range provided by the testing entity.

3) In the course of a clinical study, it is imperative that all observations and findings are thoroughly verified to ensure the reliability of the data. This guarantees that any conclusions drawn within the study are firmly rooted in raw data. Additionally, appropriate data management measures should be consistently implemented throughout both the clinical study and the subsequent data processing stages.

4) To mitigate potential dropouts, proactive steps should be implemented to maintain the case dropout rate below 10%.

5) Based on the subject's original observation records, the investigator ensures that data is accurately (aligning with the subject's actual circumstances), comprehensively (no elements should be omitted), clearly (with neat handwriting, easily recognizable), and promptly entered into the Case Report Form (CRF).

19 Publishing of Articles

Publishing regulations:

The results of this clinical study will be published in a signed article by the investigator and affiliation (the first author and corresponding author of the article). Other personnel, based on their respective contributions to the study, are entitled to be acknowledged as co-first authors and co-corresponding author.

Reference

1. Wang Y, Zhou S, Yang F, et al. Treatment-Related Adverse Events of PD-1 and PD-L1 Inhibitors in Clinical Trials: A Systematic Review and Meta-analysis. *JAMA oncology*. Jul 1 2019;5(7):1008-1019. doi:10.1001/jamaoncol.2019.0393

2. Wan G, Chen W, Khattab S, et al. Multi-organ immune-related adverse events from immune checkpoint inhibitors and their downstream implications: a retrospective multicohort study. *The Lancet Oncology*. Aug 2024;25(8):1053-1069. doi:10.1016/s1470-2045(24)00278-x

3. Thompson LL, Krasnow NA, Chang MS, et al. Patterns of Cutaneous and Noncutaneous Immune-Related Adverse Events Among Patients With Advanced Cancer. *JAMA dermatology*. May 1 2021;157(5):577-582. doi:10.1001/jamadermatol.2021.0326

4. Thompson JA, Schneider BJ, Brahmer J, et al. Management of Immunotherapy-Related Toxicities, Version 1.2022, NCCN Clinical Practice Guidelines in Oncology. *Journal of the National Comprehensive Cancer Network : JNCCN*. Apr 2022;20(4):387-405. doi:10.6004/jnccn.2022.0020

5. Duong TA, Valeyrie-Allanore L, Wolkenstein P, Chosidow O. Severe cutaneous adverse reactions to drugs. *Lancet (London, England)*. Oct 28 2017;390(10106):1996-2011. doi:10.1016/s0140-6736(16)30378-6

6. Goodman RS, Johnson DB, Balko JM. Corticosteroids and Cancer Immunotherapy. *Clinical cancer research : an official journal of the American Association for Cancer Research*. Jul 14 2023;29(14):2580-2587. doi:10.1158/1078-0432.Ccr-22-3181

7. Phillips GS, Wu J, Hellmann MD, et al. Treatment Outcomes of Immune-Related Cutaneous Adverse Events. *Journal of clinical oncology : official journal of the American Society of Clinical Oncology*. Oct 20 2019;37(30):2746-2758. doi:10.1200/jco.18.02141

8. Quach HT, Johnson DB, LeBoeuf NR, Zwerner JP, Dewan AK. Cutaneous adverse events caused by immune checkpoint inhibitors. *Journal of the American Academy of Dermatology*. Oct 2021;85(4):956-966. doi:10.1016/j.jaad.2020.09.054

9. Xue C, Yao Q, Gu X, et al. Evolving cognition of the JAK-STAT signaling pathway: autoimmune disorders and cancer. *Signal transduction and targeted therapy*. May 19 2023;8(1):204. doi:10.1038/s41392-023-01468-7
10. Duggan S, Keam SJ. Upadacitinib: First Approval. *Drugs*. Nov 2019;79(16):1819-1828. doi:10.1007/s40265-019-01211-z
