# Supplementary material for: Upadacitinib for Immune Checkpoint Inhibitor–Related Dermatitis: A Nonrandomized Clinical Trial
Source: JAMA Oncol. 2026 Mar 5;12(5):526–8. doi: 10.1001/jamaoncol.2026.0136 (PMC12964249; doi:10.1001/jamaoncol.2026.0136)
Supplement: Supplement 2. — Statistical Analysis Plan [file jamaoncol-e260136-s002.pdf]

_

JAK inhibitors for Immune Checkpoint Inhibitors-Related Dermatitis: An Open-Label, Single-Arm, Phase II Clinical Trial

Statistical Analysis Plan

**Protocol No.:**	HangCH051
**Sponsor:**	The Quzhou Affiliated Hospital of Wenzhou Medical University, Quzhou People's Hospital, Quzhou, China
**Statistical Analysis Company:**	The Quzhou Affiliated Hospital of Wenzhou Medical University, Quzhou People's Hospital, Quzhou, China
**Version No.:**	V1.0
**Version Date:**	2024-11-28

Table of Contents

Statistical Analysis Plan ... 1
1. Introduction ... 3
2. Study Objectives ... 3
3. Study Design ... 3
    3.1 Overall Study Design ... 3
    3.2 Sample Size Calculation ... 3
4. Study Endpoints ... 4
    4.1 Efficacy Endpoints ... 4
5. Analysis Sets ... 5
6. Statistical Analysis ... 5
    6.2 Demographics and Baseline Characteristics ... 7
    6.3 Efficacy Analyses ... 7
    6.4 Pharmacodynamics Analyses ... 7
    6.5 Safety Analyses ... 7

1. Introduction

The document provided is the Statistical Analysis Plan (SAP) for the study titled "JAK inhibitors for Immune Checkpoint Inhibitors-Related Dermatitis: An Open-Label, Single-Arm, Phase II Clinical Trial". The SAP outlines the statistical analysis methods for demographic and characteristics, efficacy and safety evaluation. Specific statistical analysis methods are proposed for each relevant endpoint, taking into account the characteristics outlined in the protocol and the study's specific requirements. The SAP will be finalized and approved before the database is locked. Minor adjustments to the statistical analysis methods may occur based on unforeseeable changes in the final data distribution.

2. Study Objectives

Primary Study Objective

- Evaluate the safety of JAK inhibitors (JAKi) in adult patients with ICI-related dermatitis.
- Explore the efficacy of JAKi in adult patients with ICI-related dermatitis.

Other Study Objective

- Explore the proportion of continued ICIs utilization at 28 days.
- Explore the change of pruritus severity assessed by Peak Pruritus Numerical Rating Scale (PP-NRS), score 0-10, a higher score indicates a more severe pruritus condition.

3. Study Design

3.1 Overall Study Design

This study adopts a prospective, single-arm, open-label design. 33-35 subjects will be enrolled and administered upadacitinib at a dosage of 15mg once daily for 28days.

This is a single-arm, open label study, and randomization and blinding are not applicable.

3.2 Sample Size Calculation

This is a Simon two-stage clinical trial. The historical response rate of corticosteroids is 65%, and the expected response rate is 85%, a sample size of 14 cases is planned for

enrollment in the first phase. The study will proceed to the next phase if the number of patients with effective treatment is > 10; otherwise, it will be terminated. The second phase is designed to enroll 19 patients. If the total number of effective responses exceeds 25, indicating that the treatment is effective. A 5%-10% dropout rate was considered in the sample size calculation, resulting in a total sample size of 35 subjects.

4. Study Endpoints

4.1 Efficacy Endpoints

4.1.1 Primary Efficacy Endpoints

1) The efficacy evaluated by the proportion of patients achieving relief from rashes (defined as ICI-related dermatitis grade ≤1 according to CTCAE v5.0).

2) Evaluate the safety of JAK inhibitors in adult patients with ICI-related dermatitis. The safety will be assessed based on the incidence and severity of adverse events (AEs) and serious adverse events (SAEs) during upadacitinib treatment. The severity of AEs will be graded using NCI CTCAE v5.0.

4.1.2 Secondary Efficacy Endpoints

1）Explore the proportion of continued ICIs utilization at 28 days.

2）Explore the change of pruritus severity assessed by Peak Pruritus Numerical Rating Scale (PP-NRS), score 0-10, a higher score indicates a more severe pruritus condition.

5. Analysis Sets

• **Intention-to-Treat (ITT):** Includes all enrolled subjects.

• **Full Analysis Set (FAS):** Includes all enrolled subjects who receive at least one dose of the study drug.

• **Per Protocol Set (PPS):** Subset of FAS， includes all subjects who complete the administration according to the protocol with no major protocol violation.

• **Pharmacodynamics Analysis Set (PDS）:** Includes all subjects who receive at least one dose of the study drug and have at least one valid pharmacodynamic data after treatment.

• **Safety Set (SS):** Includes all subjects who receive at least one dose of the study drug.

All above analysis sets will be discussed and decided jointly by the principal investigator, the statistician, and data management personnel at the data review meeting prior to the database lock.

6. Statistical Analysis

6.1 Basic Principles

6.1.1 General Rules

6.1.1.1 Basic Analysis

All Statistical analyses will be conducted using SAS 9.4, SPSS 26.0 or R, version 4.2.0software. Unless otherwise specified, the following rules will be adhered to in all statistical analyses:

1) For continuous data, the statistical description will include the number of observations, missing values, mean, median, standard deviation, minimum, and maximum.

2)For categorical data, the statistical description will comprise the frequency and percentage for each category.

3)All statistical tests will be two-sided with a significance level (alpha) of 0.05, and

95% confidence intervals (CI) will be utilized.

6.1.1.2 Decimal Places

The number of decimal places for each statistical parameter is presented in Table 1:

Table 1 Number of Decimal Places

Statistics	Number of decimal places
Mean, Median, Q1, Q3, CI	1 more decimal place than the raw data and not more than 3 decimal places
SD	1 more decimal place than the raw data and not more than 3 decimal places
Min, Max	The same number of decimal places as the raw data, not more than 3 decimal places
Percentage	Round to one decimal place.<br>The percentage is expressed as "100" if it is 100%, "0" if it is 0, and "-" if the denominator is 0.
P Value	If P value is ≥ 0.0001, round to 4 decimal places;<br>if P value is < 0.0001, report as < 0.0001

6.1.1.3 Baseline

The baseline is defined as the last non-missing result obtained before the administration of the first dose of the study drug (including that day).

6.1.1.4 Study Day

The study day is defined as the number of days from the first dose of the study drug to the occurrence of the event. The day of the first dose of the study drug is considered Day 1 (D0). If the date is missing or incomplete, the study day will not be calculated. The calculation of the study day is as follows:

- If the event occurs on or after the first dose of the study drug:

  Study Day = assessment date - first dose of the study drug date
- If the event occurs before the first dose of the study drug:

  Study Day = assessment date - first dose of the study drug date;

6.2 Demographics and Baseline Characteristics

6.2.1 Demographics and Clinical Characteristics

Descriptive statistics will be provided for the following demographics and clinical characteristics:

- Demographics: age, sex, height, weight, and body mass index;
- Clinical characteristics:
  a. Cycle and type of ICI drugs used.
  b. Grading and classification of ICI-related dermatitis
  c. Types of cancers
  d. Presence of pruritus and PP-NRS score;

6.3 Efficacy Analyses

6.3.1 Categorical Endpoints

The number and proportion of subjects who achieved dermatitis relief (defined as ICI-related dermatitis grade ≤1 according to CTCAE v5.0) and pruritus alleviation (defined as a reduction of over 4 points according to PP-NRS) will be summarized descriptively. Additionally, the 95% confidence interval (CI) of the proportion will be calculated using Clopper-Pearson method.

6.4 Pharmacodynamics Analyses

Pharmacodynamics endpoints will be summarized descriptively.

6.5 Safety Analyses

6.5.1 Adverse Events

The number and incidence of adverse events (AEs), serious adverse events (SAEs) and AEs with different severities will be summarized, respectively.
