# Supplementary material for: Upadacitinib for Immune Checkpoint Inhibitor–Related Dermatitis: A Nonrandomized Clinical Trial
Source: JAMA Oncol. 2026 Mar 5;12(5):526–8. doi: 10.1001/jamaoncol.2026.0136 (PMC12964249; doi:10.1001/jamaoncol.2026.0136)
Supplement: Supplement 3. — Data Sharing Statement [file jamaoncol-e260136-s003.pdf]

Data Sharing Statement

Chen. Upadacitinib for Immune Checkpoint Inhibitor–Related Dermatitis. *JAMA Oncol*.
Published March 05, 2026. doi:10.1001/jamaoncol.2026.0136

Data
Additional Information: ClinicalTrials.gov number, NCT06715982
Data available: Yes
Data types: Deidentified participant data
How to access data: wushixiu@medmail.com.cn
When available: With publication

Supporting Documents
Document types: Statistical/analytic code
How to access documents: wushixiu@medmail.com.cn
When available: With publication

Additional Information
Who can access the data: researchers whose proposed use of the data has been approved
Types of analyses: for a specified purpose
Mechanisms of data availability: after approval of a proposal, or with a signed data access agreement
